# Pure Mucinous Breast Carcinoma: Integrating Molecular and Immunohistochemical Profiles for Accurate Diagnosis—A Case Report

**DOI:** 10.1155/crom/6760604

**Published:** 2026-04-18

**Authors:** Hozaifa Hendi, Hasan Asjiee, Hamza Hendi, Batoul Obeid, Ahmad Alhaj

**Affiliations:** ^1^ Faculty of Medicine, University of Aleppo, Aleppo, Syria, alepuniv.edu.sy; ^2^ Department of Pathology, Aleppo University Hospital, Aleppo, Syria, auh-orl.com; ^3^ Department of Surgery, University of Aleppo, Aleppo, Syria, alepuniv.edu.sy

## Abstract

**Background:**

Mucinous carcinoma of the breast (MCB) is a rare histological subtype accounting for approximately 2% of all breast cancers, and it is characterized by abundant extracellular mucin production, indolent clinical behavior, and a favorable prognosis compared with other invasive breast carcinomas. Early and accurate recognition is essential due to its distinct pathological and immunohistochemical features.

**Case Presentation:**

A 50‐year‐old woman with no significant medical or family history presented with a palpable mass in her right breast, so she underwent breast screening. The imaging revealed multiple irregular nodular lesions with clustered microcalcifications in the right breast (BI‐RADS 4C), so she underwent a right modified radical mastectomy with axillary dissection. However, histopathological examination confirmed a well‐differentiated pure mucinous carcinoma (> 90% mucinous component, Nottingham Grade I) without lymphovascular invasion or nodal metastasis (0/13). The tumor was ER/PR–positive, HER2‐negative, with a low Ki‐67 index (3%), and was completely excised with negative margins.

**Discussion:**

Pure MCB exhibits favorable biological behavior and excellent outcomes, particularly when accurately diagnosed through histopathological and immunohistochemical evaluation.

**Conclusion:**

Pure MCB is a rare, low‐grade tumor with distinct pathological features. Accurate diagnosis and complete surgical excision are essential for optimal management, as patients typically achieve excellent long‐term outcomes.

## 1. Introduction

Breast cancer is the most common female gynecological cancer, with more than 800,000 new cases diagnosed worldwide [1]. However, mucinous carcinoma (MC) of the breast is a rare histological type that is characterized by a large amount of mucin production, and it is defined as having a mucinous component of 50% or more [2]. MC can be classified into two principal subtypes according to the amount of mucinous component: the pure type (pure mucinous carcinoma [PMC]) and the mixed type (mixed mucinous carcinoma [MMC]) [3].

In addition, MCB accounts for approximately 2% of all breast cancer cases [4]. It predominantly affects postmenopausal and elderly women [5]. MCB is characterized by a slow growth pattern and a lower propensity for lymphatic or distant metastasis [6].

Clinically, many patients present with palpable breast masses; however, a significant proportion exhibit nonpalpable mammographic abnormalities detected during routine screening [7].

Herein, we present a case of MC of the breast that was surgically excised, emphasizing its clinical presentation and pathological findings.

The work has been reported in line with SCARE criteria [8].

## 2. Case Presentation

A 50‐year‐old female patient, with no history of chronic illness or family history of breast cancer, presented with a palpable mass in the right breast during a routine breast‐imaging visit.

Gross examination revealed multiple well‐circumscribed, gelatinous nodular masses, the largest measuring approximately 3 × 2 × 2 cm.

**Figure 1:**
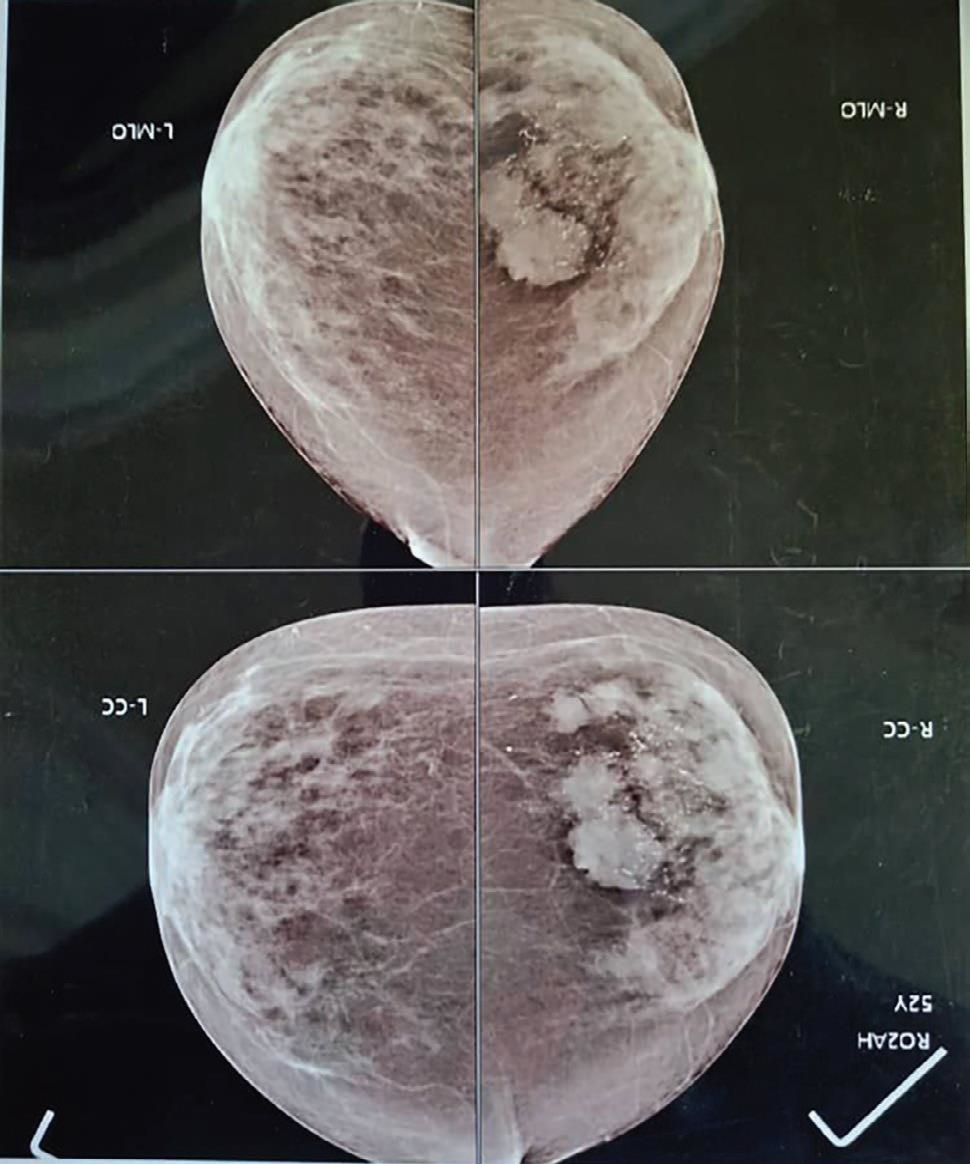
Mammography of both breasts showing the right breast with clustered microcalcifications and irregular nodular masses.

Bilateral mammography in craniocaudal (CC) and mediolateral oblique (MLO) showed that the right breast had a heterogeneously dense parenchymal pattern, with an average distribution of glandular and fibrous tissue. Clustered punctate microcalcifications with a suspicious appearance were noted, along with four nodular masses measuring between 1 and 3 cm in diameter. These lesions were located in the upper and lower inner quadrants of the right breast and exhibited irregular margins (Figure 1).

Ultrasonographic evaluation demonstrated a heterogeneous internal echotexture. There was no nipple retraction, skin thickening, parenchymal distortion, or axillary lymphadenopathy. Based on these findings, the lesion was classified as BI‐RADS 4C. Due to the central location of the mass and the patient′s small breast size, coupled with a high clinical suspicion of malignancy, the decision was made to proceed directly with surgery without a preoperative biopsy.

The patient underwent a modified radical mastectomy of the right breast with axillary lymph node dissection, during which 13 axillary lymph nodes were identified.

**Figure 2:**
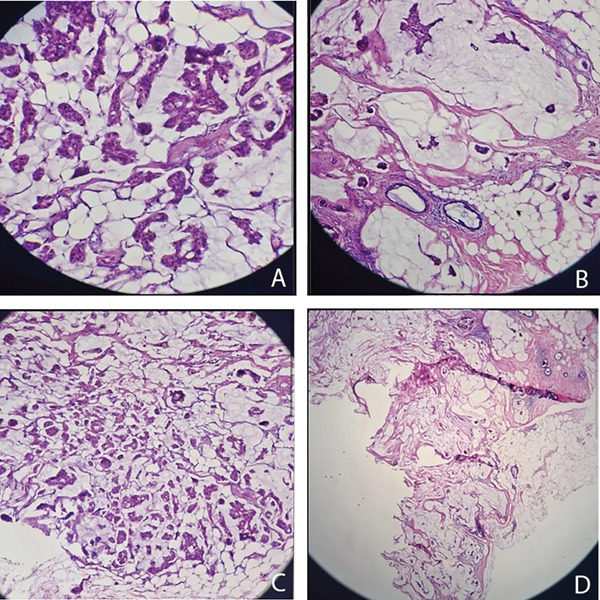
Histopathologic image showing invasive mucinous carcinoma with abundant extracellular mucin.

Microscopic examination of multiple nodular lesions revealed a malignant epithelial proliferation composed of well‐differentiated glands and cribriform structures floating in abundant extracellular mucin, representing more than 90% of the tumor volume. The tumor cells showed enlarged hyperchromatic nuclei, prominent nucleoli, and occasional atypical mitotic figures, surrounded by reactive fibrous stroma. The adjacent breast tissue showed fibrocystic changes (Figure 2).

Histologic grading according to the Scarff–Bloom–Richardson (SBR)/Nottingham system revealed tubular/glandular differentiation of Grade I, nuclear pleomorphism of Grade II, and mitotic count of Grade I, corresponding to an overall Grade I invasive carcinoma. No lymphovascular invasion was identified. The nipple, pectoral fascia, overlying skin, and all surgical margins were free of malignancy. All 13 axillary lymph nodes showed reactive changes with no metastatic involvement (0/13).

**Figure 3:**
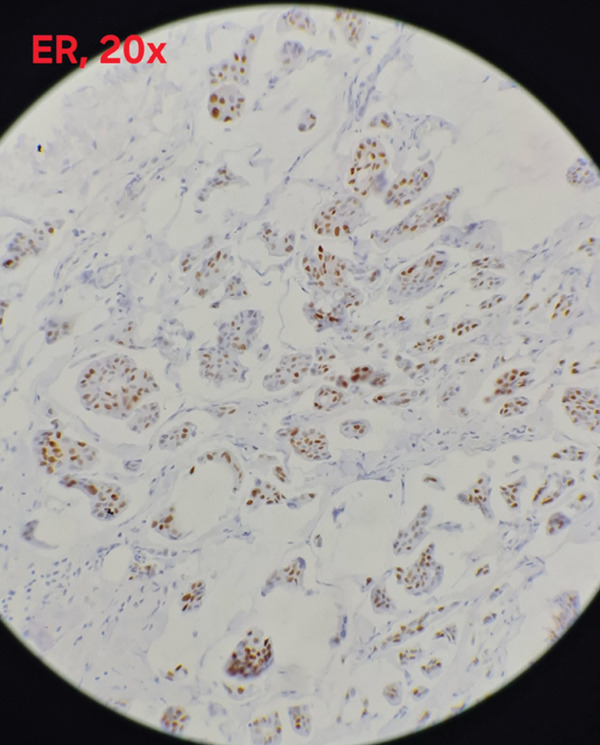
Estrogen receptor (ER) nuclear positivity (Allred score 6/8) showing strong diffuse nuclear staining in tumor cells (×20; scale bar = 50 *μ*m).

**Figure 4:**
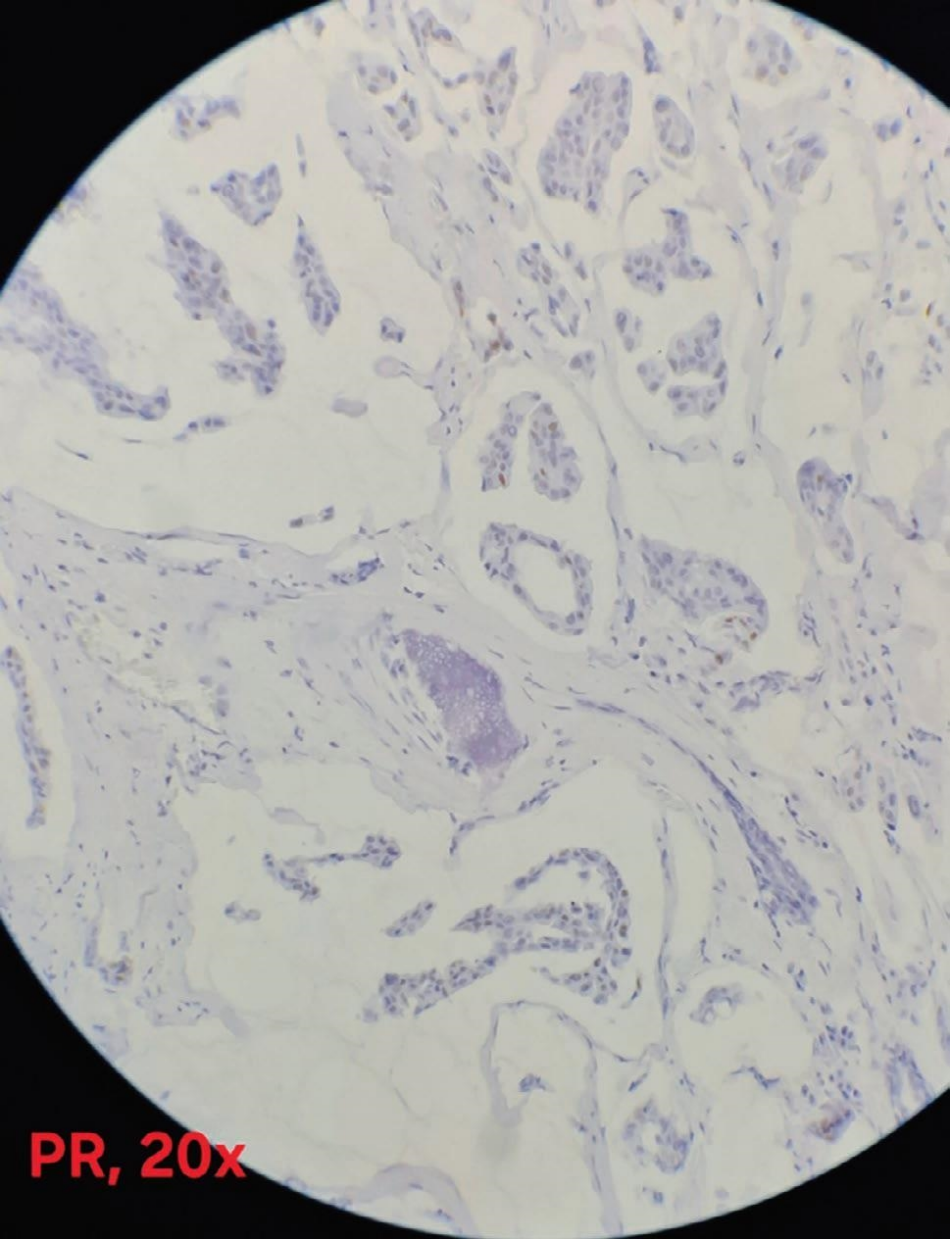
Progesterone receptor (PR) with focal/moderate nuclear staining (Allred score 3/8) (×20; scale bar = 50 *μ*m).

**Figure 5:**
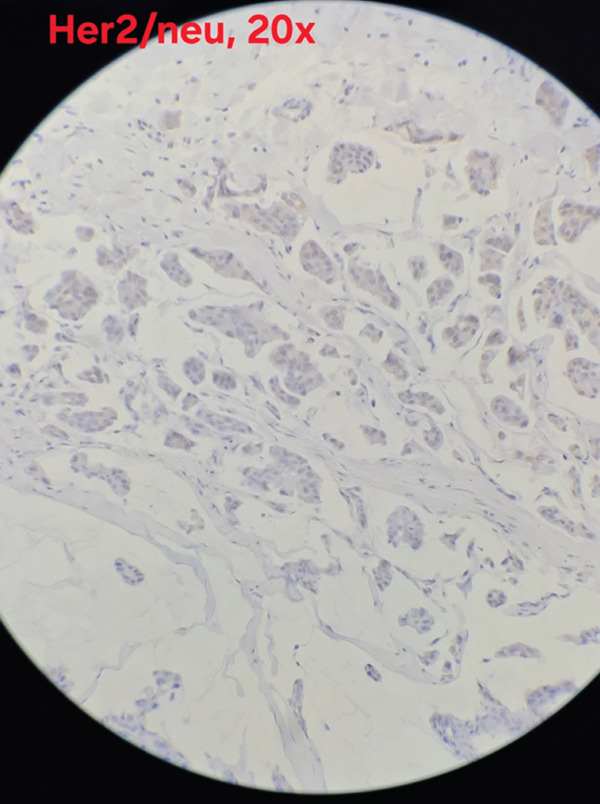
HER2/neu immunostaining demonstrating absence of membranous overexpression (score 0) (×20; scale bar = 50 *μ*m).

**Figure 6:**
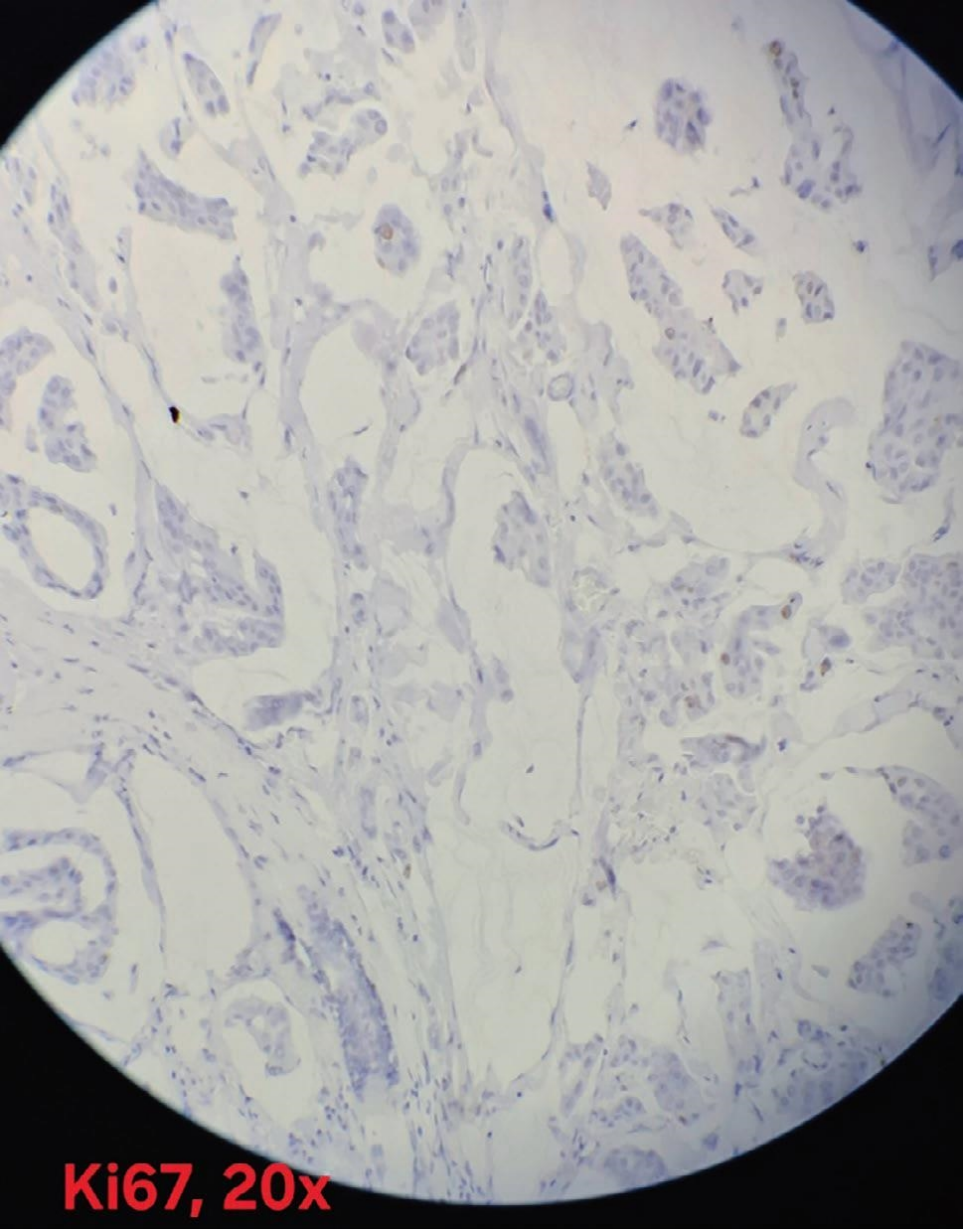
Ki‐67 proliferation index low (~3%), showing a rare positive nuclei consistent with low proliferative activity (×20; scale bar = 50 *μ*m).

Immunohistochemical analysis showed estrogen receptor (ER) positivity with an Allred score of 6/8 (Figure 3), and progesterone receptor (PR) expression was positive with an Allred score of 3/8 (Figure 4). HER2/neu was negative (score 0) (Figure 5), and the Ki‐67 proliferation index was low (3%)(Figure 6).

Based on the clinical, histopathological, and immunohistochemical findings, a final diagnosis of multifocal invasive MC of the right breast, Grade I, was established following modified radical mastectomy. There was no evidence of lymphovascular invasion or nodal metastasis, and the tumor was completely excised. The patient was scheduled to begin complementary therapy under the care of the oncologist, following the institutional treatment protocol.

## 3. Discussion

Mucinous (colloid) carcinoma of the breast represents one of the rarer subtypes of breast neoplasms [9]. This tumor type is generally associated with a low risk of lymph node involvement; however, the likelihood of nodal metastasis tends to increase with larger tumor size and in mixed histologic variants [10, 11]. Additionally, MCs are classified into pure and mixed types based on extracellular mucin content [12]: pure when the mucin content exceeds 90%, and mixed when the mucin content ranges between 75% and 90%, although the precise cutoff remains a subject of debate [13]. As observed in the present case, where approximately 90% of the lesion was composed of mucinous material, this corresponds to a PMC.

Histologically, these tumors exhibit clusters of uniform epithelial cells floating in large pools of extracellular mucin, separated by delicate fibrous septa. The tumor cells are typically small, with hyperchromatic nuclei and inconspicuous nucleoli [1]. Because the assessment of mucin proportion requires evaluation of the entire tumor, this classification can only be accurately determined from surgical excision specimens rather than limited core biopsy samples [4]. In the present case, preoperative imaging classified the breast lesion as BI‐RADS 4C. Given the central location of the mass, the patient′s small breast size, and the high clinical suspicion of malignancy, the following management options were discussed with the patient: ultrasound‐guided core needle biopsy with immunohistochemical analysis, a process estimated to require approximately 6 weeks for final results, or proceeding directly to mastectomy. The patient elected to undergo mastectomy to expedite her therapeutic course. Consequently, surgical intervention was performed without prior biopsy.

Although MC of the breast most commonly presents as a solitary lesion, a multifocal pattern, as demonstrated in our case on both mammographic and ultrasonographic imaging, can also be encountered [14]. PMC typically appears as an oval or round lesion with isodense to hyperdense characteristics and circumscribed or microlobulated margins [5], with a soft to firm consistency related to its high mucin content, whereas MMC lesions are generally firmer on palpation [15, 16]. However, up to 20% of MCs may not be visualized mammographically and can instead present as calcifications or focal asymmetries [17].

Immunohistochemical analysis of MC typically reveals positivity for ER (94%) and PR (80%), with negative HER2 expression in approximately 93% of cases [14–18], findings generally associated with a favorable prognosis [19].

In our case, the immunohistochemical profile was HER2‐negative, ER‐positive, and PR‐positive. The tumor also demonstrated a low Ki‐67 proliferation index, a parameter known to correlate with favorable outcomes and delayed recurrence in breast cancer [20]. Therefore, evaluation of Ki‐67 remains an important prognostic factor in the management and follow‐up of such patients.

## 4. Limitations

A significant aspect of this case that warrants discussion is the administration of adjuvant chemotherapy. While the patient received a TC (docetaxel and cyclophosphamide) regimen based on the treating oncologist′s clinical judgment—considering her age (50 years) and tumor size (3 cm)—this decision diverges from current evidence‐based guidelines. The tumor′s favorable biological profile (pure mucinous histology, Grade I, node‐negative status, strong hormone receptor positivity, HER2 negativity, and a Ki‐67 index of 3%) classifies it as a luminal A‐like subtype, for which the addition of chemotherapy to endocrine therapy offers negligible benefit and is not routinely recommended.

## 5. Conclusion

MC of the breast is a rare histologic subtype characterized by abundant extracellular mucin and generally associated with a favorable prognosis. Accurate diagnosis requires comprehensive histopathological evaluation and immunohistochemical characterization. The present case highlights the clinical course and histologic features of PMC. Awareness of this uncommon entity is essential for accurate diagnosis, appropriate management, and prognostic evaluation. Surgical excision remains the cornerstone of therapy, and patients typically achieve excellent outcomes when complete resection is accomplished.

## Funding

No funding was received for this manuscript.

## Ethics Statement

This study is exempt from ethical approval in our institution because the content of the case report does not require ethical approval

## Consent

Written informed consent was obtained from the patient to publish this case report and any accompanying images.

## Conflicts of Interest

The authors declare no conflicts of interest.

## Data Availability

The data that support the findings of this study are available on request from the corresponding author. The data are not publicly available due to privacy or ethical restrictions.

## References

[1] Bencherifi Y. , Watik F. , Lyafi Y. , Mostapha B. , Ennachit M. , and Mohammed E. K. , Mucinous Carcinoma of the Breast: Rare Histological Entity to Know: About Two Cases and Review of Literature, International Journal of Surgery Case Reports. (2023) 110, 108652, 10.1016/j.ijscr.2023.108652, 37579629. 37579629 PMC10448262

[2] Bae S. Y. , Choi M. Y. , Cho D. H. , Lee J. E. , Nam S. J. , and Yang J. H. , Mucinous Carcinoma of the Breast in Comparison With Invasive Ductal Carcinoma: Clinicopathologic Characteristics and Prognosis, Journal of Breast Cancer. (2011) 14, no. 4, 308–313, 10.4048/jbc.2011.14.4.308, 2-s2.0-84863067777, 22323918. 22323918 PMC3268928

[3] Sugawara-Komatsu K. , Fujii T. , Kurozumi S. , Ishikawa H. , Katayama A. , Handa T. , Tougou M. , Sano T. , Matsumoto H. , Kurosumi M. , Horiguchi J. , Shirabe K. , and Oyama T. , Significance of MUC1 and *β*-Catenin Localization in Mucinous Carcinoma of the Breast, Anticancer Research. (2024) 44, no. 6, 2689–2698, 10.21873/anticanres.17076, 38821605. 38821605

[4] Jung M. , Mucinous Carcinoma of the Breast: Distinctive Histopathologic and Genetic Characteristics, Kosin Medical Journal. (2022) 37, no. 3, 176–186, 10.7180/kmj.22.022.

[5] Hamdy O. , Shetiwy M. , Saber M. M. , Eldawody B. A. , Kassab S. A. , Nabih M. H. , Abdelhakiem M. , Zaki M. , Yussif S. M. , Saleh S. , and Abdelwahab K. , Mucinous Carcinoma of the Breast: Epidemiological, Clinical, and Prognostic Characteristics; A Single-Center Experience, Discover Oncology. (2025) 16, no. 1, 10.1007/s12672-025-03146-2, 40702277. PMC12287483 40702277

[6] Wang S. , Zhang Y. , Yin F. , Zhang X. , Yang Z. , and Wang X. , Prognostic Analysis of Primary Breast Signet Ring Cell Carcinoma and Mucinous Breast Adenocarcinoma: A SEER Population-Based Study, Frontiers in Oncology. (2021) 10, no. 11, 783631, 10.3389/fonc.2021.783631, 34956901. PMC8702493 34956901

[7] Prajapati Y. H. , Bhabhor V. , Mehta K. S. , Barot M. , Boriwala H. , and Omar M. , Pure Mucinous Adenocarcinoma of the Breast With the Rare Lymphoplasmacytic Infiltration: A Case Report With Review of Literature, Clinical Case Reports. (2024) 12, no. 4, e8560, 10.1002/ccr3.8560, 38550744. 38550744 PMC10965451

[8] Sohrabi C. , Mathew G. , Maria N. , Kerwan A. , Franchi T. , Agha R. A. , and Collaborators , The SCARE 2023 Guideline: Updating Consensus Surgical Case Report (SCARE) Guidelines, International Journal of Surgery. (2023) 109, no. 5, 1136–1140, 10.1097/JS9.0000000000000373, 37013953. 37013953 PMC10389401

[9] Lacroix-Triki M. , Suarez P. H. , MacKay A. , Lambros M. B. , Natrajan R. , Savage K. , Geyer F. C. , Weigelt B. , Ashworth A. , and Reis-Filho J. S. , Mucinous Carcinoma of the Breast Is Genomically Distinct From Invasive Ductal Carcinomas of No Special Type, Journal of Pathology. (2010) 222, no. 3, 282–298, 10.1002/path.2763, 2-s2.0-77958022477, 20815046. 20815046

[10] Dumitru A. , Procop A. , Iliesiu A. , Tampa M. , Mitrache L. , Costache M. , Sajin M. , Lazaroiu A. , and Cirstoiu M. , Mucinous Breast Cancer: A Review Study of 5 Year Experience From a Hospital-Based Series of Cases, Maedica. (2015) 10, no. 1, 14–18, 26225144. 26225144 PMC4496759

[11] Chtourou I. , Makni S. K. , Bahri I. , Abbes K. , Sellami A. , Fakhfakh I. , Gouiaa N. , Ayadi L. , Frikha M. , Daoud J. , and Boudawara T. S. , Carcinome colloïde pur du sein: étude anatomoclinique de sept cas [Pure Colloid Carcinoma of the Breast: Anatomoclinical Study of Seven Cases], Cancer/Radiothérapie. (2009) 13, no. 1, 37–41, 10.1016/j.canrad.2008.06.004, 2-s2.0-58249128374, 18703371. 18703371

[12] Anwar S. L. , Dwianingsih E. K. , Avanti W. S. , Choridah L. , Suwardjo A. T. , and Aryandono T. , Aggressive Behavior of Her-2 Positive Colloid Breast Carcinoma: A Case Report in a Metastatic Breast Cancer, Annals of Medicine and Surgery. (2020) 52, no. 52, 48–52, 10.1016/j.amsu.2020.02.010, 32211189. 32211189 PMC7082430

[13] Di Saverio S. , Gutierrez J. , and Avisar E. , A Retrospective Review With Long-Term Follow-Up of 11,400 Cases of Pure Mucinous Breast Carcinoma, Breast Cancer Research and Treatment. (2008) 111, no. 3, 541–547, 10.1007/s10549-007-9809-z, 2-s2.0-51649096934, 18026874. 18026874

[14] Ha K. Y. , Deleon P. , and Deleon W. , Invasive Mucinous Carcinoma of the Breast, Baylor University Medical Center Proceedings. (2013) 26, no. 3, 295–297, 10.1080/08998280.2013.11928989, 23814397. 23814397 PMC3684304

[15] Achicanoy Puchana D. M. , Lasso Andrade F. A. , Achicanoy Puchana D. F. , Boada Fuentes M. A. , Álvarez Duarte M. A. , Angarita Acuña K. , Jaime Aguirre A. C. , Muñoz Murillo J. A. , González Lago A. M. , Alegria Cuellar D. A. , Orozco Morales L. K. , Lasso Anacona M. Z. Z. , Alvarado Rengifo A. E. , and Rosero Rosero J. R. , Mucinous Carcinoma of the Breast: Diagnosis and Management of an Unusually Young Patient, Radiology Case Reports. (2022) 17, no. 5, 1445–1449, 10.1016/j.radcr.2022.02.011, 35265238. 35265238 PMC8899128

[16] Bode M. and Rissanen T. , Imaging Findings and Accuracy of Core Needle Biopsy in Mucinous Carcinoma of the Breast, Acta Radiologica. (2011) 52, no. 2, 128–133, 10.1258/ar.2010.100239, 2-s2.0-79958858851, 21498339. 21498339

[17] Marrazzo E. , Frusone F. , Milana F. , Sagona A. , Gatzemeier W. , Barbieri E. , Bottini A. , Canavese G. , Rubino A. O. , Eboli M. G. , Rossetti C. M. , Testori A. , Errico V. , De Luca A. , and Tinterri C. , Mucinous Breast Cancer: A Narrative Review of the Literature and a Retrospective Tertiary Single-Centre Analysis, Breast. (2020) 49, 87–92, 10.1016/j.breast.2019.11.002, 31783314. 31783314 PMC7375663

[18] Thai J. N. , Lerwill M. F. , and Chou S. S. , Spectrum of Mucin-Containing Lesions of the Breast: Multimodality Imaging Review With Pathologic Correlation, Radiographics. (2023) 43, no. 10, e230015, 10.1148/rg.230015, 37792588. 37792588

[19] Lei L. , Yu X. , Chen B. , Chen Z. , and Wang X. , Clinicopathological Characteristics of Mucinous Breast Cancer: A Retrospective Analysis of a 10-Year Study, PLoS One. (2016) 11, no. 5, e0155132, 10.1371/journal.pone.0155132, 2-s2.0-84973121257, 27232881. 27232881 PMC4883756

[20] Nishimura R. , Osako T. , Okumura Y. , Hayashi M. , Toyozumi Y. , and Arima N. , Ki-67 as a Prognostic Marker According to Breast Cancer Subtype and a Predictor of Recurrence Time in Primary Breast Cancer, Experimental and Therapeutic Medicine. (2010) 1, no. 5, 747–754, 10.3892/etm.2010.133, 2-s2.0-77955203082, 22993598. 22993598 PMC3445951

